# Cut-off points to screening for sarcopenia in community-dwelling older people residents in Brazil

**DOI:** 10.7717/peerj.12038

**Published:** 2021-08-25

**Authors:** Sabrina Gabrielle Gomes Fernandes, Luiz Eduardo Lima de Andrade, Rafaella Silva dos Santos Aguiar Gonçalves, Saionara Maria Aires da Câmara, Ricardo Oliveira Guerra, Alvaro Campos Cavalcanti Maciel

**Affiliations:** 1 Postgraduate Program in Physiotherapy, Federal University of Rio Grande do Norte, Natal, Rio Grande do Norte, Brazil; 2 Postgraduate Program in Rehabilitation Sciences. Faculty of Health Sciences of Trairi, Federal University of Rio Grande do Norte, Santa Cruz, Rio Grande do Norte, Brazil

**Keywords:** Epidemiology, Cut-off points, Population specific, Prevalence, Sarcopenia

## Abstract

**Background:**

At a time when the world’s population is aging, one of the most important challenges for the healthcare field is to control the decline of the musculoskeletal system. This decline consists of a reduction in muscle mass and function, which is called sarcopenia and is associated with adverse health outcomes. Although there has been an increase in the number of publications on sarcopenia and its consequences, the reported prevalence varies widely, since these depend on the characteristics of the population studied, the definitions found in the literature and the cut-off points adopted. In this perspective, the heterogeneity in the classification and the different reference values has a critical impact on the epidemiology of sarcopenia, since neither the procedures, the components and the cut-off points are consistent.

**Objectives:**

To develop cut-off points for the screening of sarcopenia in community-dwelling older people residents in the northeast of Brazil and compare the prevalences between the values defined by the consensus and the values of the population studied.

**Methods:**

Community-dwelling older men and women living in three cities in the countryside of Rio Grande do Norte were evaluated. Cutoff points were defined for the variables used to screen for sarcopenia (handgrip strength, SMI, gait speed and SPPB) using the 20th percentile of their population distributions.

**Results:**

The sample was composed of 1,290 older people (62.5% female and 37.5% male), with an average of 69.5 (± 6.05) years of age. Regarding the cutoff points, the handgrip values were defined as 25.3 kg and 16 kg for men and women, respectively. Considering the SMM adjusted according to their height, the values of 7.88 kg/m^2^ were adopted for men and 5.52 kg/m^2^ for women. When adjusting by BMI we obtained 0.73 kg/BMI for men and 0.41 kg/BMI for women. For gait speed it was defined 0.71 m/s for men and 0.63 m/s for women. In the case of SPPB, the result was the same for both genders (≤8). When applying the values found in the studied population, a variation in prevalence was observed for both men and women, depending on the cut-off points and consensus used.

**Conclusion:**

The cutoff values found in our population were lower than those adopted by international consensus (EWGSOP2, IWGS and FNIH), except for HGS in woman and SMI/m^2^ for men. Therefore, using specific cutoff points for different populations can provide an accurate assessment of the presence of sarcopenia and better target health prevention strategies for the older people living in the community.

## Introduction

At a time when the world’s population is aging, one of the most important challenges for the healthcare field is to control the decline of the musculoskeletal system (Chen et al., 2014). This decline consists of a reduction in muscle mass and function, which is called sarcopenia (Chen et al., 2014) and is associated with adverse health outcomes such as disability, worse quality of life and falls (Cruz-Jentoft et al., 2010; Yang et al., 2020). Due to its relevance in the field of gerontology and geriatrics, since 2018, sarcopenia is considered a muscle disease and is formally included in the International Classification of Diseases (Cruz-Jentoft et al., 2019).

Currently, several definitions have been proposed by different study groups to standardize the conceptual approach to sarcopenia (Cruz-Jentoft et al., 2010; Chen et al., 2014; Studenski et al., 2014). In 2010, the European Working Group on Sarcopenia in Older People (EWGSOP) published a consensus, widely used worldwide, which provides a definition and screening strategy for diagnosing and evaluating sarcopenia (Cruz-Jentoft et al., 2010). This definition was revised in 2018 in order to propose a new operational definition and a clinical algorithm, recommending the criteria of low muscle strength and low mass to diagnose sarcopenia (Yang et al., 2020).

In addition to the EWGSOP, other groups of experts have made efforts to determine operational criteria for this diagnosis, such as the International Working Group on Sarcopenia (IWGS), which proposed that the diagnosis of sarcopenia should include low skeletal muscle mass and low physical performance (Fielding et al., 2011). The National Institutes of Health (FNIH) also consider muscle weakness and low lean mass as criteria for the diagnosis of sarcopenia (Studenski et al., 2014).

Although there has been an increase in the number of publications on sarcopenia and its consequences, the reported prevalence varies widely (Bahat et al., 2018; Erdogan et al., 2021), since these depend on the characteristics of the population studied, the definitions found in the literature and the cut-off points adopted (Cruz-Jentoft et al., 2010; Fielding et al., 2011; Studenski et al., 2014; Chen et al., 2014; Cruz-Jentoft et al., 2019; Yang et al., 2020). The literature (von Haehling, Morley & Anker, 2010; Tonial et al., 2020; Petermann-Rocha et al., 2020; Van Ancum et al., 2020) already shows that this prevalence can vary between 10–50%, and in Brazil this variation is 1–29% for elderly community members (Diz et al., 2017).

In this perspective, the heterogeneity in the classification and the different reference values has a critical impact on the epidemiology of sarcopenia, since neither the procedures, the components and the cut-off points are consistent (Viana et al., 2018). The cut-off points adopted by the consensus depend on the measurement techniques and availability of studies and populations (Cruz-Jentoft et al., 2010), recommending the use of normative data instead of predictive reference populations (Bahat et al., 2016), thus evidencing the need to obtain cut-off points for populations worldwide (Cruz-Jentoft et al., 2010).

Except for Viana et al. (2018) and Sampaio et al. (2017), who developed cut-off points for the screening of sarcopenia in elderly community members in southeastern Brazil, the number of studies that present the same proposal is still scarce in the country, especially for those elderly who live in the Brazilian northeast. Therefore, elderly people living in this region are diagnosed through the cut-off points of international consensus that do not consider the different ethnic characteristics of Brazil, which may lead to an overestimation of the prevalence of sarcopenia in this population (Sampaio et al., 2017). Unlike other areas, the northeast region is considered one of the poorest places in Brazil, characterized by high rates of illiteracy, child mortality and unemployment (Ponciano, Nunes & Cerdeira, 2015), in addition to presenting high rates of social inequalities that affect individuals’ access to health care (Fernandes et al., 2020).

In view of the above, this work has as main objective to develop cut-off points for the screening of sarcopenia in elderly community residents in northeastern Brazil. In addition, a comparison will be made between the prevalences found considering the cut-off points already established in international consensus (Fielding et al., 2011; Studenski et al., 2014; Cruz-Jentoft et al., 2019) and the new values developed for the studied population.

## Methods

This is an observational cross-sectional study carried out in Natal, Parnamirim and Santa Cruz, all of which are located in Rio Grande do Norte. Natal is the capital of the state and has approximately 800 thousand inhabitants, Parnamirim is located in the metropolitan region of Natal and has approximately 202,456 residents, while Santa Cruz is located in the countryside of the state and has a population of 36,660 residents.

The present study is a secondary analysis of the data from three studies, where the first aimed to investigate the decline in mobility, considering the perspective of the course of life (International Mobility in Aging Study—IMIAS) (Zuzunegui et al., 2015); the second study aimed to examine the relationship between hormone levels and physical performance in middle-aged and elderly women from northeastern Brazil (da Câmara et al., 2015); and third one proposed to implement a global geriatric assessment system based on a digital platform for decision making, monitoring and promoting active aging (PRO-EVA). It should be noted that in both studies, the procedures adopted for the collection of information related to sarcopenia followed the same protocol, ensuring the quality of the data generated.

### Population and sample

The study population consisted of older men and women aged 60 and over who lived in the three cities mentioned above. The sample was chosen because of convenience, and it was obtained through disclosure in the Basic Healthcare Units (UBS). Individuals with neurological or orthopedic diseases that compromised the measurements of physical performance tests were identified, and those with cognitive alterations that hindered comprehension during the evaluations, with four or more errors in Leganès Cognitive Test (LCT) (De Yebenes et al., 2003) were also identified. Altogether, 1,290 older people were considered for the present study.

### Procedures

All volunteers were evaluated by trained interviewers at the UBSs in Parnamirim, at the Integrated Center for Teaching, Research and Community Actions (NIPEC) and at the Faculty of Health Sciences of Trairi (FACISA), campus of the Federal University of Rio Grande do Norte (UFRN), in Santa Cruz. In the case of the IMIAS study, all assessments were carried out at the participant’s home. The data were collected using a standardized protocol described below:

### Components of sarcopenia

(a) Handgrip strength (HG):

Measured using the Saehan^®^ dynamometer that provides the record of muscle strength in kilograms (kg). Participants were positioned as recommended by the American Society of Hand Therapists (Fess, 1992), seated, shoulders adducted and in neutral rotation, elbow positioned at 90° of flexion, with forearms and wrist in neutral or slightly extended position (up to 30 degrees). Participants were instructed to squeeze the dynamometer at maximum isometric strength for five seconds three times, with a one-minute interval between attempts (da Câmara et al., 2015; Fernandes et al., 2020). For this study, the average of the three attempts was considered for the analysis.

(b) Skeletal Muscle Mass Index (SMI):

Skeletal muscle mass (SMM) total was estimated using the predictive equation of Visvanathan et al. (2012), where SMM = 10.05 + (0.35 * Weight) − (0.62 * BMI) − (0.02 * Age) + 5.10 (if men). This equation showed a strong correlation with the DXA X-ray dual emission absorption measurements (*r*^2^ = 0.868) (Visvanathan et al., 2012). To find the SMI, the SMM was adjusted for the height of the participant (Cruz-Jentoft et al., 2010), and following the recommendation of the FNIH it was also adjusted using the Body Mass Index (BMI) (Studenski et al., 2014; Bahat et al., 2020).

(c) Gait speed (GS):

It was measured according to the Short Physical Performance Battery (SPPB) protocol (Guralnik et al., 1994). Participants were instructed to walk 4 m twice and the time for each attempt was recorded. For this study, gait speed was calculated by dividing the space covered (4 m) by the shortest time, in seconds.

### Physical performance

Physical performance was assessed using the SPPB (Guralnik et al., 1994). This tool includes three tests that assess the function of the lower limbs: orthostatic balance test in three positions of increasing difficulty, a 4-m walk and five repetitions of the sit-up test. Scores range from 0 to 12 and the highest score means greater physical performance. For this study, individuals who scored less than eight were classified as having a worse physical performance (Pirkle et al., 2014).

### Definition of sarcopenia

For the present study, sarcopenia was defined using the cut-off points and algorithms of the three consensuses: (I) EWGSOP2 (Cruz-Jentoft et al., 2019); (II) FNIH (Studenski et al., 2014) and (III) IWGS (Fielding et al., 2011). Subsequently, the same algorithms for decision making were used, however, the cut-off points for the specific population were applied. Although the EWGSOP2 suggested the use of muscle mass-2SD of the young reference population, this study applied the cut-off points identified by the 20th percentile for the specific population. Those with low muscle strength and low muscle quality were considered confirmed sarcopenia. The cut-off points for each consensus are summarized in Table 1.

**Table 1:** Original cut-off points according to consensus.

Consensus	Variables	Men	Women
EWGSOP2	Grip strength	<27 kg	<16 kg
	SMI/m^2^ Index	<7 kg/m^2^	<5.5 kg/m^2^
	SPPB	<8	
FNIH	Grip strength	<26 kg	<16 kg
	SMI/BMI Index	<0.78 kg/IMC	<0.51 kg/IMC
IWGS	Gait speed	1 m/s^2^	
	SMI/m^2^ Index	<7.23 kg/m^2^	<5.68 kg/m^2^

**Note:**

EWGSOP2, European Working Group on Sarcopenia in Older People; FNIH, Foundation for the National Institutes of Health; IWGS, International Work Group of Sarcopenia; SMI, Index Skeletal Muscle Mass; SPPB, Short Physical Performance Battery; BMI, Body Mass Index.

### Potential confounders

(a) Sociodemographic and anthropometric variables:

Participants were assessed for sociodemographic data such as gender (male and female), age, education, stable union and ethnicity/race. The age of the participants was collected through self-report and verified through an identification document; schooling was assessed by the number of years the participant attended school and was categorized as: none, 1–3 years, 4–7 years and 8 years or more. For the stable union status, the participants’ self-report was considered, being dichotomized as yes or no.

For anthropometric variables, height (m) and weight (kg) were measured, and BMI (kg/m^2^) was calculated, following the formula BMI = weight/height^2^ and then categorized into: normal weight (18.5 to 24.99 kg/m^2^), overweight (25.00–29.99 kg/m^2^) and obese (≥30.00 kg/m^2^).

(b) Lifestyle habits:

The participants were asked about the consumption of alcoholic beverages and tobacco, for both variables the answers were dichotomized as yes or no. As for the practice of physical activity, the participants were asked if they were practicing sports, exercises, or other physical activities, such as walking, in their leisure time, at least three times a week and for 30 min or more and it was dichotomized in yes or no (da Câmara et al., 2015; Fernandes et al., 2020).

## Ethical aspects

All participants were informed about the research objectives and procedures and at the first contact they signed the consent form. The study protocol received approval from the UFRN Ethics and Research Committee (approval numbers: 623/11, 1.875.802 and 2.996.329).

## Data analysis

The data were analyzed using the Statistical Package for Social Science (SPSS) 20.0 software. First, descriptive statistics were presented using mean ± standard deviation (SD) for normal continuous variables and non-normal continuous variables were expressed using median (interquartile range), for quantitative variables absolute and relative frequencies were used (Yang et al., 2020). To identify the cut-off values in the population studied, the 20th percentile was used for the variables of grip strength, SMI, gait speed and SPPB. A 95% confidence interval (95% CI) and a *p* value of 0.05 were adopted.

## Results

Table 2 shows the characteristics of the sample, which included 1,290 older people with an average 69.54 (± 6.05) years of age and composed mostly of women (62.5%). A greater proportion of the population attended between 4–7 years of formal education (32.3%), live in consensual union (59.3%) and are overweight (44.6%). As for life habits, most of the population was non-smoker, non-consumer of alcoholic beverages and a non-practitioner of physical activity.

**Table 2:** Sample characteristics (*N* = 1,290).

Variables			*N* (%) or Median (Q25–Q75)
Gender		Men	484 (37.5%)
		Women	806 (62.5%)
Age			69.0 (65.0–73.0)
Education	None		281 (21.8%)
	1–3 years		326 (25.3%)
	4–7 years		417 (32.3%)
	≥8 years		266 (20.6%)
Consensual union	Yes		765 (59.3%)
	No		525 (40.7%)
BMI^§^	Normal		340 (26.8%)
	Overweight		567 (44.6%)
	Obese		364 (28.6%)
BMI^§^			27.46 (24.55–30.48)
Smoker^§^	Yes		208 (16.3%)
	No		1069 (83.7%)
Consumption of alcoholic drink^§^	Yes		148 (11.6%)
	No		1129 (88.4%)
Physical activity^§^	Yes		480 (37.6%)
	No		797 (62.4%)
Physical performance (SPPB)	≥8		937 (72.6%)
	≤8		353 (27.4%)

**Notes:**

BMI, Body Mass Index; SPPB, Short Physical Performance Battery.

§ >10 missing value.

Table 3 shows the cut-off points found for the population of the present study by the 20th percentile according to gender. Considering the total sample, the prevalence of confirmed sarcopenia varied from 2.9% to 17.2%, using the original cut-off points of each consensus (EWGSOP2: 2.9%; FNIH: 10.6% and IWGS: 17.2%). However, when applying the values developed by the 20th percentile specific to our population, there is a reduction in prevalence, especially when using the algorithms referring to the IWGS (5.1%) and FNIH (4.7%).

**Table 3:** Cut-off points for community-dwelling older people (20th percentile).

Variables	Man	Woman
Grip strength	25.33 kg	16.00 kg
SMI/m^2^ Index	7.88 kg/m^2^	5.52 kg/m^2^
SMI/BMI Index	0.73 kg/IMC	0.41 kg/IMC
Gait Speed	0.71 m/s^2^	0.63 m/s^2^
SPPB	≤8	≤8

**Note:**

SMI, Skeletal Muscle Mass; M, meters; BMI, Body Mass Index; SPPB, Short Physical Performance Battery.

**Figure 1:**
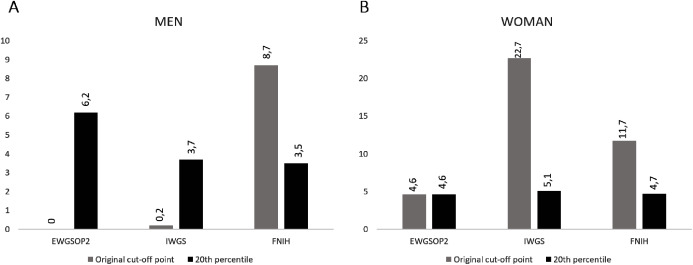
Prevalence (%) of sarcopenia in older people according to the cut-off points established in the consensus and by the 20th percentile. (A) Prevalence for men and (B) prevalence for women.

Using the original cut-off points, women had a higher prevalence of confirmed sarcopenia in the EWGSOP2 (4.6%), IWGS (27.5%) and FNIH (11.7%) consensus when compared to men (EWGSOP2: 0%, IWGS: 0.2% and FNIH: 8.7%). When applying the cut-off values found for the studied population, men presented a higher prevalence of sarcopenia only for the criteria of the EWGSOP2 (6.2%) *vs* 4.6% for women. For the FNIH and IWGS consensus, women remained with a higher prevalence of sarcopenia (4.7% and 5.1%, respectively) when compared to men (FNIH: 3.5% and 3.5%) (Figs. 1A and 1B).

**Figure 2:**
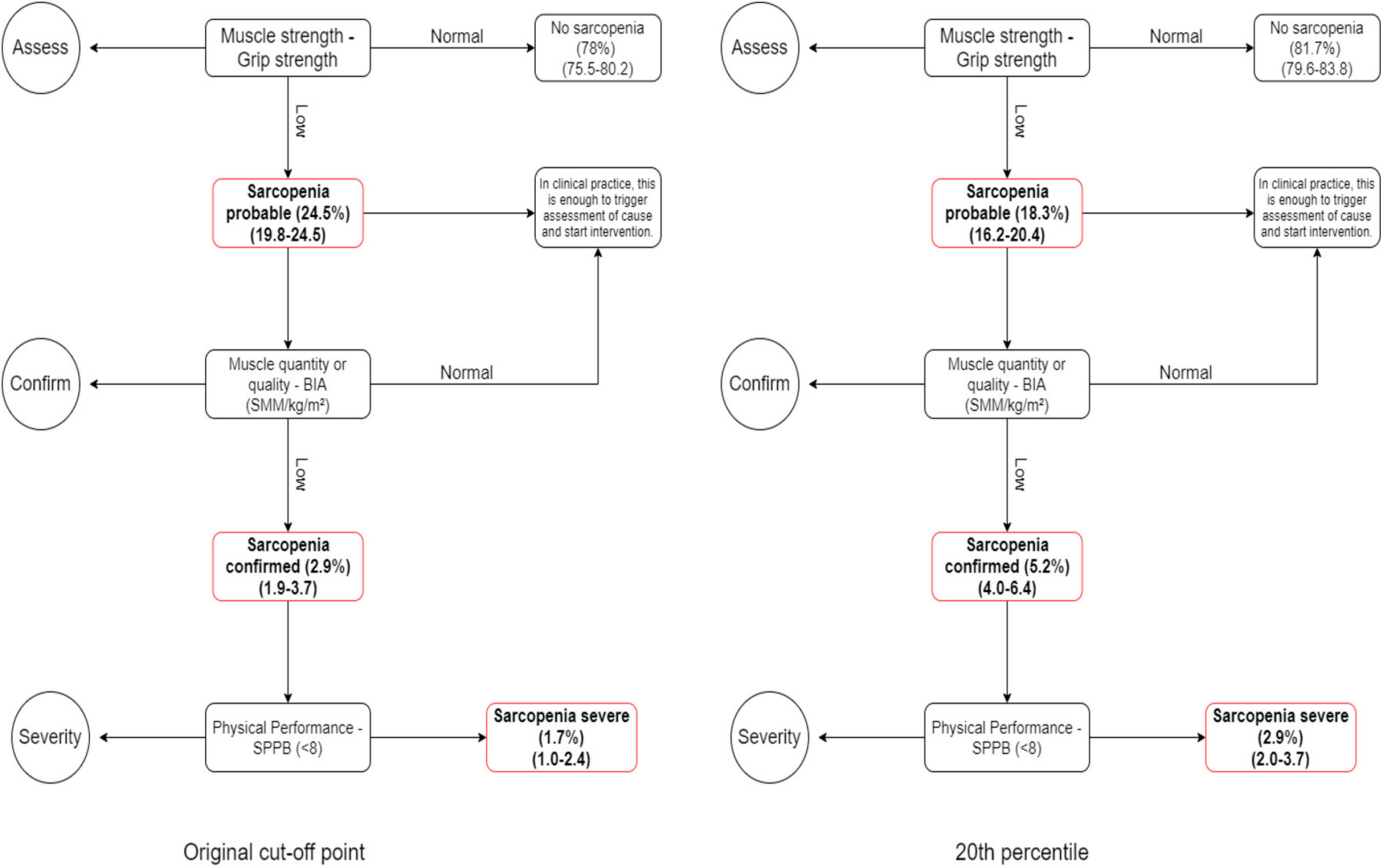
Comparison between EGWSOP2 algorithm and 20th percentile.

**Figure 3:**
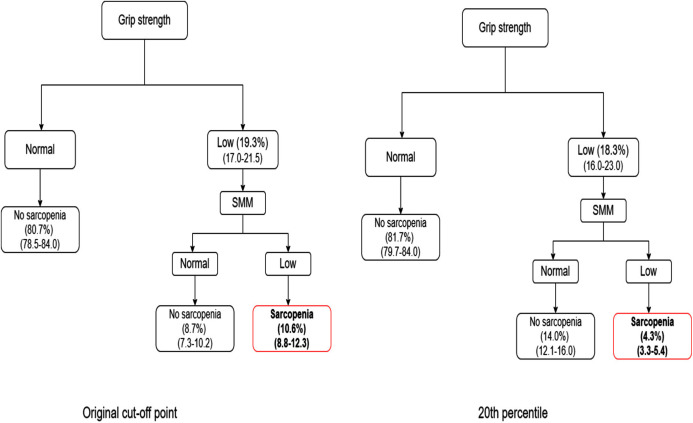
Comparison between FNIH algorithm and 20th percentile.

**Figure 4:**
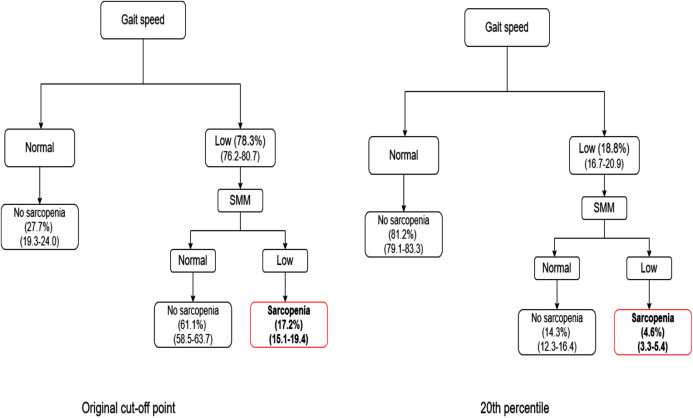
Comparison between IWGS algorithm and 20th percentile.

The Figs. 2–4 show the algorithms for each consensus with their cut-off points and those proposed by this study. In each step of the algorithm, the prevalence and confidence interval (CI) of the older people who were with or without the deficit in each criterion are presented.

## Discussion

The present study defined cut-off points for screening for sarcopenia aimed at the population residing in northeastern Brazil. As with other findings in the literature, in our study there was a predominance of females (62.5%). This result is consistent with the fact that women are more present in the search for medical assistance in Primary Health Care (PHC) (Sousa, Silver & Griep, 2010), the place where the evaluations were carried out.

Regarding the prevalence of sarcopenia, there was a variation depending on the cut-off point and the consensus used, thus reinforcing the hypothesis that the classification of sarcopenia is dependent on the method used, the chosen cut-off point, as well as the population studied (Bijlsma et al., 2013; Bahat et al., 2018, 2021).

The cut-off points for handgrip strength, gait speed and SMM have been widely used to diagnose sarcopenia in populations residing in developed countries, where they were originally defined, so using such values in other populations can lead to inaccurate prevalence rates (Moreira, Perez & Lourenço, 2019). A systematic review carried out in Brazil showed that the prevalence of sarcopenia can vary from 1–29% in the community-dwelling older people (Diz et al., 2017; Viana et al., 2018) and most of the studies found use the cut-off points proposed by the EWGSOP (Diz et al., 2017), which can induce a high prevalence rate of sarcopenia in community-dwelling older Brazilian.

When applying the original cut-off points of the three chosen consensus (Cruz-Jentoft et al., 2019; Studenski et al., 2014; Fielding et al., 2011) the prevalence varied from 2.9% to 17.2%, as observed by Bahat et al. (2018) who found a prevalence of 1.9% when using the IWGS consensus and 9.2% when using the FNIH. Unlike Bahat et al. (2018) who found a lower prevalence of sarcopenia according to the IWGS, our study showed a higher prevalence considering this consensus (17.2%). This fact corroborates the study by Yang et al. (2020) who found a higher prevalence of sarcopenia in older Chinese people, when using the IWGS consensus compared to the EWGSOP2 and FNIH.

According to Yang et al. (2020), the IWGS criteria have a lower consistency with the EWGSOP2 and FNIH, possibly due to the different conceptual structures that they present. While the EWGSOP and the FNIH take muscle strength into account, by assessing hand grip and muscle quality, adjusted for height and BMI, respectively, the IWGS classifies individuals with low muscle mass and gait speed <1.0 m/s as sarcopenic. Therefore, this high value for classifying individuals with low gait speed may have overestimated the prevalence of sarcopenia in the elderly in this sample.

When using the cut-off points developed specifically for the studied population, the prevalence of sarcopenia decreased significantly in two consensuses (IWGS = 4.6% and FNIH = 4.3%). Among the genders, a higher prevalence of sarcopenia in men can be observed when using the diagnostic criteria of the EWGSOP2 (5.2%), similar to the findings of Shafiee et al. (2020) who observed a higher prevalence of sarcopenia in this population group when compared to women, using the criteria of EWGSOP 1 and EWGSOP2.

It is important to reiterate that using the same cut-off values for populations with different genotypes and phenotypic characteristics can produce unreliable results, therefore, overestimating or underestimating the true prevalence (Arango-Lopera et al., 2012). For the studied population, the cut-off points were identified using the 20th percentile and it was found values that were lower than those found in the studied consensus (Fielding et al., 2011; Studenski et al., 2014; Cruz-Jentoft et al., 2019) except for handgrip strength in women and for SMI in men.

Regarding HG, the values of 25.3 kg and 16 kg were established for men and women, respectively. Such values differ from what was observed in studies carried out in several countries (Han et al., 2009; Shim et al., 2013), for example, Yoo, Choi & Ha (2017) evaluated Korean older people and found cutoff values of 28.8 kg for men and 18.2 kg for women. However, our results were similar to the findings in a cohort of Chilean older people (Lera et al., 2015) who have characteristics similar to those of the Brazilian population, reinforcing the hypothesis that the cutoff points found are closely related to the demographic characteristics of the population.

Associated with this, the method chosen for the evaluation of HGS, based on the American Society of Hand Therapists (Fess, 1992) and using an average of three evaluations, may have contributed to this difference. In a systematic review carried out by Ha et al. (2018) it can be observed that there are several ways to measure HGS in epidemiological studies, therefore, the lack of a consensus for this assessment contributes to heterogeneous cut-off values.

Observing the values according to gender, it is noted that for men, the cut-off point was slightly lower when compared to that established by EWGSOP2 and FNIH, similar to the findings by Shafiee et al. (2020) who found values lower than that established in the EWGSOP2. This fact can be explained because both consensuses developed their reference values based on a young and healthy population (Cruz-Jentoft et al., 2019; Studenski et al., 2014).

For women, the cut-off point for grip strength was the same as that observed in studies carried out in Europe, North America and Asia (Cruz-Jentoft et al., 2019; Studenski et al., 2014; Fielding et al., 2011), however, this value was lower than that proposed in studies carried out with the elderly Brazilians living in the southeast region of the country (Sampaio et al., 2017; Confortin et al., 2017). Naturally, women tend to have less handgrip strength due to the menopausal transition period and subsequent decline in estrogen, which can play a role in the loss of muscle mass and strength (da Câmara et al., 2015).

Associated to this, women of our population, residing in the Northeast of Brazil, accumulate adversities throughout the course of their lives, such as exposure to violence, discrimination, lower wages, low education and gender inequality that can have a significant impact on the physical performance (Guralnik et al., 1994), thus leading to lower results in the handgrip test, when compared to those women who live in other parts of the country.

When calculating the cut-off points for the total SMM, the values of 7.88 kg/m^2^ and 5.49 kg/m^2^ were established for men and women, respectively, with the values for the male sex being higher than the points proposed in the EWGSOP2 and IWGS. However, our results differ from that found by Viana et al. (2018) who evaluated elderly residents in Minas Gerais and obtained reference values for the SMM of <6.47 kg/m^2^ for women and <8.76 kg/m^2^ for men.

This result can be explained due to the ethnic, cultural, social and lifestyle differences of the populations (Dorosty et al., 2016; Papadopoulou, 2020). The low socioeconomic conditions, low education and the adversities throughout the life course in which the residents of the northeast live can have significant impacts on the skeletal muscle mass, generating results inferior to those of other regions of Brazil (Dorosty et al., 2016). Associated with this, physical inactivity is also a contributory factor for the reduction of SMM in both sexes, increasing the chances of the emergence of sarcopenia (Lourenço et al., 2014). In addition, the methodology used for identified cut-off points in this study was different who used in other studies, such as −2SD (Cruz-Jentoft et al., 2019) and regression tree analysis (CART) (Studenski et al., 2014), corroborating for this difference.

Regarding gait speed, the values found were 0.75 m/s for men and 0.66 m/s for women, these being less than 0.8 m/s, which is considered the value of reference for slow gait, except in the IWGS. Such results corroborate the findings by Moreira, Perez & Lourenço (2019) who identified cut-off points for an elderly population living in Rio de Janeiro ranging from 0.65 to 0.73 m/s for men and 0.60 to 0.69 m/s for women, adjusted for height. According to Lourenço et al. (2014), in populations in Latin America, the clinically relevant values for gait speed may differ from 0.8 m/s and the use of this value to classify the elderly with slow gait may result in bias.

This divergence between the cut-off values for gait speed can be explained by the sensitivity that this measure has in relation to racial/ethnic differences and anthropometry (Sims et al., 2009), and it can also differ significantly based on the individual’s gender and height (Bohannon & Andrews, 2011). Therefore, the elderly in the Northeast who have shorter stature and shorter lower limbs have a lower gait speed when compared to those who live in other regions of the country (Ponciano, Nunes & Cerdeira, 2015).

In addition, the low level of physical activity and muscle weakness related to age may preferentially affect the lower extremity, directly compromising the muscle performance of the elderly, significantly affecting gait speed and leading to its reduction (Henwood & Taaffe, 2005).

## Strengths and Limitations

One of the strengths of the present study was the definition of reference values that take into account the individual and regional characteristics of the people who reside in the northeastern region of Brazil, being the first study with this objective, emphasizing the need to create a specific cut-off point for each population, and consequently minimizing possible overestimation of the prevalence of sarcopenia. Furthermore, the values obtained by the method chosen was derived from the older population, unlike other studies using young populations by reference.

Another strength of this study presented is that with the cut-off points found for this population, it is possible that health professionals, especially those inserted in a context of primary health care (PHC), are able to identify the elderly who are at risk of developing sarcopenia at an early stage, making possible the elaboration of preventive measures to the aggravations resulting from the sarcopenia.

The study also had important limitations, first, there may have been a sample selection bias, with relatively healthy individuals recruited who were able to go to the assessment sites in comparison with other elderly people. Second, a prediction equation was used for the calculation of the SMM, which is not considered a gold standard for this assessment and its accuracy can be affected by several factors that compromise the interpretation of these results, however, the results agree with other research conducted in Brazil (Viana et al., 2018; Sampaio et al., 2017).

## Conclusion

The values of the specific cut-off points developed for our population differ from international consensus. Therefore, using specific cut-off points for different populations can provide an accurate assessment of the presence of sarcopenia and better target health prevention strategies for the older people living in the community.

It was concluded, therefore, that developing methods that facilitate the diagnosis of sarcopenia are crucial for both the population and healthcare professionals, especially in places such as northeastern Brazil, enabling the early identification of this important affection and enabling the design of interventions and policies to minimize the negative health effects of sarcopenia.

## Supplemental Information

10.7717/peerj.12038/supp-1 Supplemental Information 1 study database.

## Additional Information and Declarations

### Data Availability

The following information was supplied regarding data availability: The raw measurements are available in the Supplemental File.
